# Late-Onset Pseudohypoparathyroidism: A Case Report

**DOI:** 10.7759/cureus.93475

**Published:** 2025-09-29

**Authors:** Rasha Ebrahim, Noorbina Peedika Parambil, Harris Poolakundan, Adeniyi Dauda Sonibare, Feroz Jenner Poolakundan

**Affiliations:** 1 Department of Medical Education, Hamad Medical Corporation, Doha, QAT; 2 Department of Nephrology, Kunhitharuvai Memorial Group of Charities (KMCT) Medical College, Kozhikode, IND; 3 Department of Internal Medicine, Hamad Medical Corporation, Doha, QAT; 4 Department of Endocrinology, Hamad Medical Corporation, Doha, QAT

**Keywords:** adult onset, hypocalcemia, parathyroid hormone resistance, pseudohypoparathyroidism type 1b, pseudohypoparathyroidism type 2

## Abstract

Pseudohypoparathyroidism (PHP) is a rare endocrine disorder due to end-organ resistance to parathyroid hormone (PTH), resulting in hypocalcemia and hyperphosphatemia despite elevated PTH levels. It usually presents in children with distinctive features known as Albright Hereditary Osteodystrophy (AHO). We report a 33-year-old man who presented with wrist pain, muscle spasms, and tingling in his hands. Laboratory investigations revealed hypocalcemia, hyperphosphatemia, and elevated serum PTH, with normal serum magnesium, renal function, and vitamin D levels, suggestive of PTH resistance. The patient was managed with calcium and vitamin D supplementation. This case highlights the diagnostic challenges of adult-onset PHP in the absence of classical features of AHO and emphasizes the importance of recognizing biochemical patterns of PTH resistance. Timely diagnosis and management can lead to symptom resolution and prevent long-term complications, such as skeletal abnormalities, neurological symptoms, cataracts, and dental abnormalities.

## Introduction

Pseudohypoparathyroidism (PHP) is a group of rare endocrine disorders caused by end-organ resistance to parathyroid hormone (PTH) [1]. It is a rare endocrine disorder with an unknown global prevalence rate. In a study conducted in Poland over 10 years, the prevalence was reported to be 0.62 to 0.64 per 100,000 people [2]. Interestingly, multiple studies have noted increased prevalence among females when compared to males. In Poland, 64.5% of adult PHP cases were women, while in Denmark, the rate was even higher at 72% [2,3]. PHP is often diagnosed in childhood due to its association with a distinctive pattern of physical features known as Albright Hereditary Osteodystrophy (AHO). These features include short stature, obesity, round facies, brachydactyly, and ectopic ossifications of soft tissue [4]. Diagnosis is typically straightforward when such features coexist with biochemical abnormalities. However, in adults without these classical findings, the diagnosis of PHP may be delayed.

The PTH is involved in serum calcium and phosphate homeostasis by promoting bone resorption, increasing renal calcium reabsorption, reducing phosphate reabsorption, and enhancing intestinal calcium absorption through the activation of vitamin D [5]. In PHP, these mechanisms are disrupted, leading to hypocalcemia and hyperphosphatemia, despite elevated PTH [6,7]. Additionally, there is reduced activation of vitamin D, further reducing calcium absorption from the gut. The disorder primarily affects the proximal renal tubules while sparing skeletal tissue responsiveness [8]. Interestingly, PTH sensitivity in the distal tubules is preserved, which protects against hypercalciuria and nephrolithiasis, features that are seen in classical hypoparathyroidism [9].

At the molecular level, PTH resistance is caused by impaired signaling through the PTH receptor, which is coupled to the G protein stimulatory alpha subunit (Gsα). In healthy individuals, PTH binds to its receptors and activates a pathway to produce cyclic adenosine monophosphate (cAMP), which eventually results in increased calcium levels and reduced phosphate levels in the serum. In PHP, this signaling cascade is disrupted, resulting in impaired hormone response. In approximately 80-90% of PHP cases, a genetic basis can be identified. These typically involve either sporadic or autosomal dominant heterozygous inactivating mutations in the guanine nucleotide binding protein, alpha-stimulating complex (GNAS) gene, or epigenetic modifications affecting the GNAS locus on chromosome 20q13.2-q13.3 [4,8,10].

PHP is classified into five types (PHP1A, PHP1B, PHP1C, PHP2, and Pseudopseudohypoparathyroidism (PPHP)) based on phenotype, hormone resistance profile, cAMP response to exogenous PTH, and Gsα protein activity [9]. In 2016, the European Pseudohypoparathyroidism Network (EuroPHP) network proposed a broader classification, ‘inactivating PTH/PTHrP signaling disorder (iPPSD)’ to unify PHP and other related conditions [11].

Our case reports the late presentation of PHP in an adult with symptomatic hypocalcemia with elevated PTH levels, hyperphosphatemia, and hypocalciuria, in the absence of classical features of AHO. This case highlights the diagnostic challenges of PHP in adults, especially when physical features are absent and symptoms are subtle. Furthermore, adult-onset cases of PHP remain underreported in the literature, particularly in the Middle East, making this case a valuable contribution to regional clinical awareness.

## Case presentation

A 33-year-old man presented to the emergency department with complaints of tingling in both hands and feet and generalized muscle cramps for 3 days. He didn’t have any episodes of seizure or stridors. One year before this presentation, he experienced similar symptoms and was admitted for four days, during which he received intravenous (IV) calcium, magnesium, and vitamin D. He was discharged on oral cholecalciferol, calcium carbonate, and magnesium, to which he was compliant. He was also diagnosed with hypothyroidism one year ago, and he was on levothyroxine. He has no history of malabsorptive syndromes, renal failure, liver disease, chronic pancreatitis, or infertility. He was born to non-consanguineous parents and has no family history of hypocalcemia or unexplained death. He has no history of surgery or irradiation to the neck.

On arrival at the emergency department, he was vitally stable. Physical examination revealed round faces and obesity (BMI 30.02 kg/m^2^) and carpopedal spasms. There was no Archibald’s sign, murderer’s thumb, no subcutaneous calcifications, cataracts, or dental hypoplasia. His laboratory investigations demonstrated low adjusted calcium (1.57 mmol/L, normal: 2.20-2.60), hyperphosphatemia (1.52 mmol/L, normal: 0.80-1.50), normal magnesium level, mild hypokalemia (3.3mmol/L, normal: 3.5-5.3), normal thyroid function tests, high parathyroid hormone level (129 pg/mL, normal: 15-65), normal 25-hydroxyvitamin D (55ng/mL) and normal serum creatinine. All other laboratory investigations were within normal range (Table 1). Electrocardiogram (ECG) demonstrated sinus bradycardia, no QT prolongation, and the chest X-ray was normal. He was given IV calcium gluconate and oral potassium chloride. On re-evaluation, his serum calcium improved to 1.73 mmol/L and adjusted calcium to 1.69 mmol/L, and his symptoms resolved.

**Table 1:** Results of laboratory investigations in the emergency department.

Parameter	Patient’s Value	Normal Range
White Blood Cells (WBC)	7.1 × 10^3^/µL	4.0-10.0
Red Blood Cells (RBC)	5.1 × 10^6^/µL	4.5-5.5
Hemoglobin (Hb)	15.5 g/dL	13.0-17.0
Hematocrit (Hct)	44.7 %	40.0-50.0
Platelet	339 × 10^3^/µL	150-410
Urea	2.7 mmol/L	2.5-7.8
Creatinine	94 µmol/L	62-106
Sodium	137 mmol/L	133-146
Potassium	3.3 mmol/L	3.5-5.3
Chloride	93 mmol/L	95-108
Bicarbonate	32 mmol/L	22-29
Calcium	1.65 mmol/L	N/A
Adjusted Calcium	1.57 mmol/L	2.20-2.60
Phosphorus	1.52 mmol/L	0.80-1.50
Magnesium	0.81 mmol/L	0.70-1.00
Total Bilirubin	11 µmol/L	0-21
Total Protein	84 g/L	60-80
Albumin	44 g/L	35-50
Alkaline Phosphatase (ALP)	94 U/L	40-129
Alanine Transaminase (ALT)	23 U/L	0-41
Aspartate Transaminase (AST)	21 U/L	0-40
Lipase	61 U/L	13-60
C-Reactive Protein (CRP)	2.2 mg/L	0.0-5.0
Thyroid Stimulating Hormone (TSH)	3.93 mIU/L	0.30-4.20
Free Thyroxine (FT4)	18.6 pmol/L	11.0-23.3
Parathyroid Hormone (PTH)	129 pg/mL	15-65
Vitamin D	55 ng/mL	N/A

Further laboratory investigations revealed spot urine potassium 41 mmol/L and a transtubular potassium gradient (TTKG) of 4.52. 24-hour urinalysis revealed hypocalciuria (1.4 mmol/24 hours, normal: 2.5-7.5). All other parameters were within the normal range (Table 2). Investigations for autoimmune disorders were negative. Anti-transglutaminase antibody was negative. Anti-thyroid peroxidase levels were high (293 IU/mL, normal: 0-34). Fecal elastase was within the normal range (>500).

**Table 2:** Results of urine chemistry.

Parameter	Patient’s Value	Normal Range
24-Hour Urine (U24) Total Volume	3,024 mL	N/A
24-Hour Urine (U24) Creatinine	12.25 mmol/24 h	9.00-21.00
24-Hour Urine (U24) Potassium	54 mmol/24 h	25-125
24-Hour Urine (U24) Total Volume	1,980 mL	N/A
24-Hour Urine (U24) Calcium	1.4 mmol/24 h	2.5-7.5
24-Hour Urine (U24) Creatinine	12.73 mmol/24 h	9.00-21.00
Urine Creatinine	11,305 µmol/L	N/A
Urine Osmolality	830 mmol/kg	150-1,150
Urine Sodium	242 mmol/L	N/A
Urine Potassium	41.0 mmol/L	N/A
Urine Chloride	35 mmol/L	N/A

**Figure 1:**
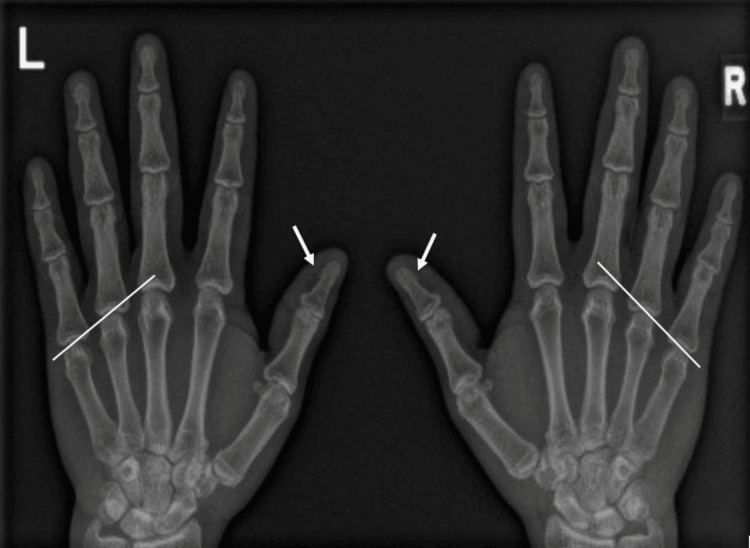
X-ray of both hands with no signs of brachydactyly, excluding AHO features. Normal hand X-ray demonstrating absence of skeletal abnormalities. A line drawn across the heads of the 4th and 5th metacarpals passes distal to the 3rd metacarpal head, indicating no metacarpal shortening. White arrows point to the distal phalanges of the thumb, showing normal bone length. AHO: Albright’s Hereditary Osteodystrophy

**Figure 2:**
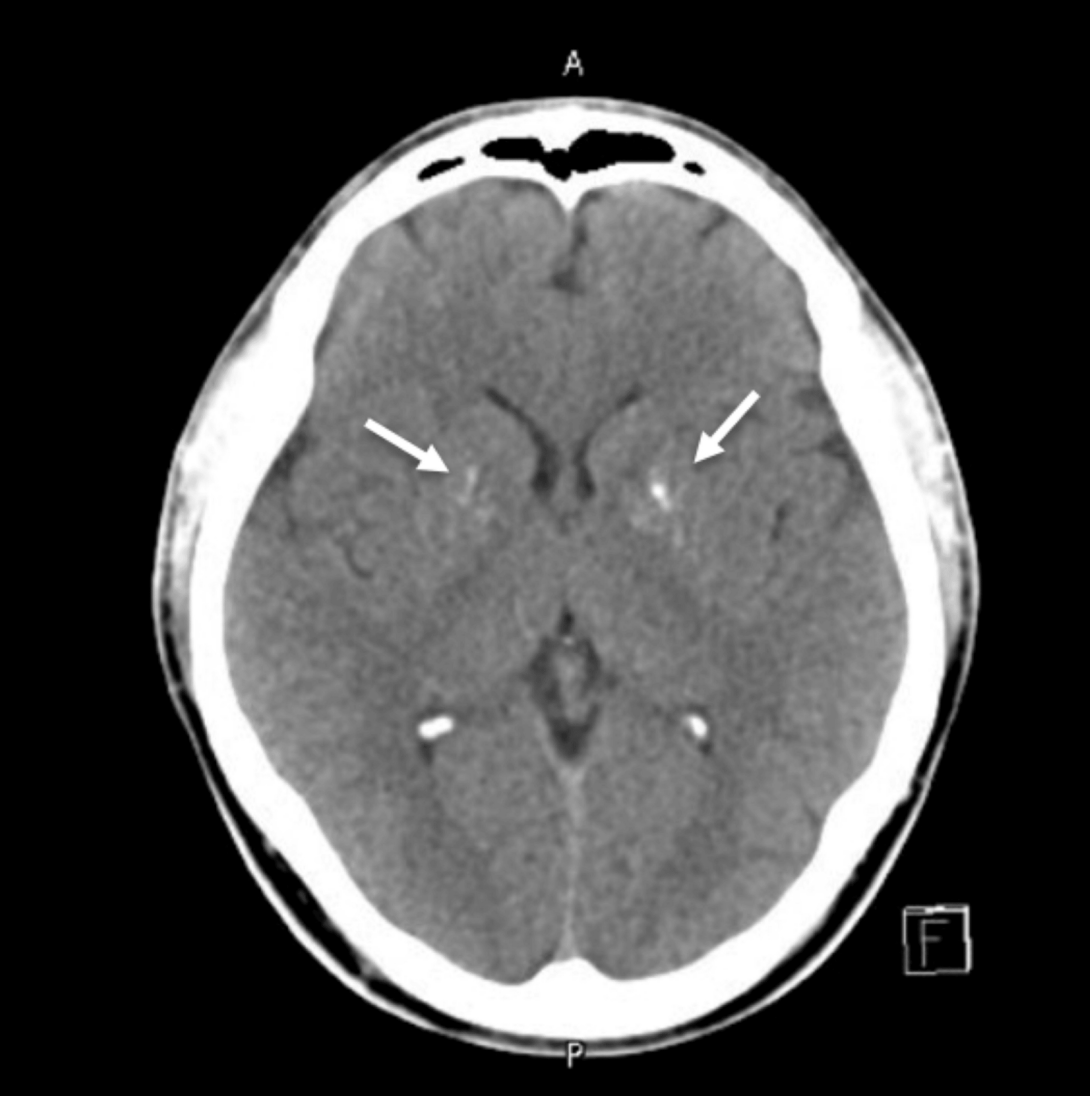
CT head demonstrating bilateral basal ganglia calcifications. White arrows point to the areas of bilateral basal ganglia calcifications.

The X-rays of both hands (Figure 1) showed no shortened metacarpal or shortened distal phalanx of the thumb, excluding any features of AHO. CT head (Figure 2) revealed bilateral basal ganglia foci of calcifications.

His inpatient medications included calcitriol 0.5 mcg once daily, calcium carbonate 1250mg thrice daily, levothyroxine 50 mcg once daily, and potassium chloride 30 mEq thrice daily. He was asymptomatic and was discharged on calcitriol 0.5 mcg twice daily, calcium carbonate 1250 mg twice daily, and levothyroxine 50 mcg once daily.

The patient was followed up after two months in the endocrinology clinic, and he was well and asymptomatic during the interval period. His laboratory investigations on follow-up demonstrated normal serum calcium levels and suppressed PTH (10 pg/mL; normal: 15-65). Furthermore, he had high anti-thyroid peroxidase (248.0 pmol/L, normal: 145.0-569.0). All other laboratory values were normal (Table 3). He underwent bone densitometry, which was normal. The patient was advised to continue the same management for maintenance, and he is scheduled to undergo an ultrasound of the thyroid and parathyroid to rule out any other underlying primary pathologies.

**Table 3:** Laboratory evaluation during follow-up.

Parameter	Patient’s Value	Normal Range
Calcium	2.50 mmol/L	N/A
Adjusted Calcium	2.50 mmol/L	2.20-2.60
Phosphorus	1.28 mmol/L	0.80-1.50
Albumin	40 g/L	35-50
Alkaline Phosphatase (ALP)	65 U/L	40-129
Creatine Kinase (CK)	134 U/L	39-308
Parathyroid hormone (PTH)	10 pg/mL	15-65
Anti-Thyroid Peroxidase (Anti-TPO)	282 IU/mL	0-34

## Discussion

Hypocalcemia is a commonly encountered electrolyte abnormality in clinical practice with a wide range of causes. It typically presents with neuromuscular symptoms such as paresthesia, tetany, or seizures, and may be associated with signs like Chvostek’s or Trousseau’s [4]. Our patient, a 33-year-old male, presented with bilateral wrist pain, muscle spasms, tingling in his hands, and carpopedal spasm on examination. His recurrent symptoms despite adherence to calcium, vitamin D, and magnesium supplementation raised suspicion for an underlying disorder of calcium regulation, such as PTH resistance.

Initial investigations revealed hypocalcemia with elevated PTH, indicating PTH resistance. However, this pattern is not exclusive to PHP. Differential diagnoses include vitamin D deficiency, chronic kidney disease, hypomagnesemia, malabsorption, autoimmune disorders, pancreatitis, and medications [1]. In this case, normal 25-hydroxyvitamin D levels (55 ng/mL) excluded vitamin D deficiency. Preserved renal function was confirmed by normal creatinine and estimated glomerular filtration rate. Magnesium, lipase, and fecal elastase were within normal limits, ruling out hypomagnesemia and pancreatitis. Autoimmune screening was negative, except for elevated anti-thyroid peroxidase antibodies. Furthermore, the absence of neck surgery and medications reduced the likelihood of secondary causes. Therefore, PHP remained the most likely diagnosis in this patient.

Most adult-onset cases of PHP reported in the literature present with seizures or long-standing neuromuscular symptoms. For instance, a case report of a 34-year-old woman presenting with seizures was reported in 2020, and another case series found that 50% of their patients presented with seizures [12,13]. Others reported prolonged histories of perioral numbness, cramps, or muscle spasms over several years [14-16]. In contrast, our patient presented with recent-onset wrist pain, spasms, and tingling without a history of seizure or prolonged symptoms. This demonstrates the variable clinical presentation in PHP and the importance of considering this diagnosis even in patients with mild symptoms.

The diagnosis of PHP is primarily based on clinical presentation and biochemical profile. According to the first consensus meeting, diagnostic criteria include PTH resistance, ectopic ossifications, early-onset obesity with TSH resistance, family history, and features of AHO. Major features of AHO include brachydactyly type E and short stature relative to the unaffected parent. Additional features include a stocky build, round faces compared to siblings, and subcutaneous ossifications. Other findings, such as obesity, dental anomalies, and cognitive impairment, may be seen, but they are not required for diagnosis [17]. Our patient did not fulfill the criteria for AHO. He had no short stature and lacked brachydactyly or skeletal abnormalities on hand X-rays. Moreover, he had no signs of ectopic ossifications, cognitive impairment, or dental anomalies.

Biochemically, PTH resistance is defined as hypocalcemia, hyperphosphatemia, and elevated serum PTH in the absence of vitamin D deficiency and with normal magnesium levels and normal renal function [17]. In this case, the patient fulfilled the criteria for PTH resistance as defined. Although the biochemical profile in PHP is consistent among cases, the clinical features vary with genetic and imprinting differences [16]. Our patient also had a history of hypothyroidism with positive anti-TPO antibodies. Although the presence of anti-TPO antibodies suggests an autoimmune etiology, it does not exclude the possibility of coexistence of GNAS-related TSH resistance. In a study by Molinaro et al., hypothyroidism preceded the development of PHP1b, suggesting that hypothyroidism could be an early sign before the development of PHP1b. Furthermore, one of the four patients studied tested positive for anti-TPO antibodies and was later diagnosed with PHP1b. In that case, the hypothyroidism was attributed to both autoimmune thyroiditis and TSH resistance caused by a GNAS mutation [18]. However, no direct association between the presence of autoimmune thyroiditis and PHP has been established in the current literature, and further research is required in this field.

Based on the classification discussed previously, the patient’s presentation is most consistent with PHP1B or PHP2 (corresponding to iPPSD3 or iPPSDx, respectively). Both of these subtypes lack AHO features and may present in adulthood. Additionally, our patient also had hypothyroidism. These features support the diagnosis of PHP1B or PHP2 clinically. The current gold standard for confirmatory diagnosis of the subtype is based on molecular testing [17]. However, in this case, we were unable to perform genetic testing to reach a conclusive diagnosis on the subtype due to financial constraints. The absence of molecular confirmation in this case represents a limitation to our study as it prevents the definitive classification of the PHP subtype, leading to uncertainty regarding long-term prognosis, potential hormonal resistances, and inheritance patterns. Without molecular diagnosis, it becomes difficult to offer accurate genetic counseling or assess the risk for other family members. Although molecular testing is the gold standard for definitive diagnosis, it may be unavailable in resource-limited settings due to cost or lack of access to specialized testing. In such situations, clinicians must rely on clinical features and biochemical findings to make a presumptive diagnosis. This is still clinically valuable as the treatment plan is similar across all PHP subtypes, and early recognition and management are crucial to prevent complications in such cases.

A comparative summary of the traditional and iPPSD classification is provided in Table 4 to illustrate the overlap in clinical and molecular features across the subtypes and support the diagnostic reasoning.

**Table 4:** Integrated Classification of Pseudohypoparathyroidism and Related Disorders. iPPSD: Inactivating Parathyroid Hormone/Parathyroid Hormone-Related Protein Signaling Disorder; PHP: Pseudohypoparathyroidism; PPHP: Pseudopseudohypoparathyroidism; AHO: Albright’s Hereditary Osteodystrophy; POH: Progressive Osseous Heteroplasia; DMRs: Differentially Methylated Regions; Gsα: Alpha Subunit of the Stimulatory G Protein; LH: Luteinizing Hormone; FSH: Follicle Stimulating Hormone; GHRH: Growth Hormone-Releasing Hormone; cAMP: Cyclic Adenosine Monophosphate [4,9,17]

Classical Classification	iPPSD Classification	Molecular Basis	Hormone Resistance	Exogenous PTH Response	Associated Syndromes/Common Clinical Features
PHP1A	iPPSD 2	Gsα mutation - GNAS Inactivating mutation in the maternal allele	PTH, TSH, LH, FSH, GHRH	Decreased cAMP Decreased Urine PO₄	AHO, POH, short stature, obesity, brachydactyly, subcutaneous ossifications, cognitive impairment
PHP1B	iPPSD 3	Imprinting dysregulation - methylation changes at one or more GNAS DMRs	PTH, TSH	Decreased cAMP Decreased Urine PO₄	Hypocalcemia symptoms, no AHO features
PHP1C	iPPSD 2	Gsα mutation - undefined; few maternal inactivating mutations	PTH, TSH, LH, FSH	Decreased cAMP Decreased Urine PO₄	Similar to PHP 1A
PHP2	iPPSDx	Undefined	PTH and variable multi-hormone resistance	Normal cAMP response Decreased Urine PO₄	Hypocalcemia symptoms
PPHP	iPPSD 2	Gsα mutation - GNAS Inactivating mutation in paternal allele	None	Normal	AHO

The patient in this case was treated according to the currently accepted management, which includes calcium supplementation and use of active vitamin D metabolites [17]. Prolonged excessive levels of serum PTH may result in elevated bone turnover and tertiary hyperparathyroidism [1]. Therefore, the goal of treatment is to maintain serum PTH within the upper normal range, promoting calcium reabsorption in the kidneys and preventing hypercalciuria and the formation of renal stones, while minimizing adverse effects on bone mineralization [17]. In this case, the patient was treated with IV calcium supplementation in the acute phase and discharged on oral calcium and vitamin D supplementation.

Complications of PHP include intracranial calcifications, cataracts, and dental anomalies. Furthermore, despite preserved skeletal responsiveness, prolonged PTH elevation can lead to bone complications such as osteitis fibrosa cystica. In rare instances, excessive urinary excretion of urinary phosphorus and uric acid may predispose patients to nephrocalcinosis [9,17]. Patients with PHP may present with neurological symptoms such as seizures and epilepsy. Other reported presentations include impaired speech, altered mental status, unsteady gait, and acute Parkinsonism [13,19,20]. Interestingly, in one case report, non-specific symptoms such as headache and diplopia prompted neuroimaging, which revealed bilateral basal ganglia calcifications, leading to further investigations and diagnosis of PHP [21]. This highlights the importance of considering PHP as a potential diagnosis in patients with basal ganglia calcifications, even if neurological symptoms are absent. In our patient, there were no neurological symptoms. However, the CT head demonstrated bilateral basal ganglia calcifications. Furthermore, bone densitometry in our patient was normal, and he did not exhibit any other complications at the time of evaluation.

Long-term management of patients with PHP involves regular follow-up and biochemical monitoring. For asymptomatic adult patients receiving treatment, it is recommended to measure serum PTH, calcium, and phosphorus levels every six months. In specific populations, such as pregnant or breastfeeding women, individuals undergoing rapid growth, those experiencing acute illness, or symptomatic patients, follow-up should be arranged more frequently as clinically indicated. During routine follow-up, physicians should conduct thorough assessments to monitor for potential complications. This includes renal imaging to detect nephrocalcinosis and CT head if neurological symptoms arise. Ophthalmological evaluation should be conducted to rule out cataracts, and regular dental follow-up should be scheduled to identify any dental anomalies early, especially in young patients. Additionally, in female patients, menstrual history should be reviewed at each visit, and if irregularities such as oligomenorrhea or amenorrhea are noted, further laboratory evaluation of gonadal function should be initiated. Similarly, male patients should be assessed for symptoms of hypogonadism. Furthermore, patients with PHP require regular assessment of metabolic and cardiovascular risk factors. This includes evaluation of BMI, dietary habits, lipid and glucose metabolism, and blood pressure control. Patients should receive tailored nutritional counseling, and they should be educated about the possibility of ectopic ossifications [17]. Our patient in this case was followed up 2 months later, and he remained asymptomatic and had no signs of any of the above-mentioned complications. His biochemical evaluations revealed normal serum calcium levels and suppressed PTH, reflecting successful treatment, and the patient was planned to continue on the same management.

## Conclusions

This case demonstrates the importance of considering PHP in adults presenting with unexplained or recurrent hypocalcemia, even when classical features of AHO are absent. It highlights the importance of clinical evaluation and biochemical criteria to aid diagnosis when genetic testing is unavailable. Although subtyping is important for diagnosis, it is not essential for treatment initiation, as the management remains consistent among all subtypes. Early recognition and appropriate management can prevent complications and improve patient outcomes.
